# Consistency and Completeness of YouTube Condom Use Instructions Compared to Health Organisation Guidelines

**DOI:** 10.7759/cureus.100367

**Published:** 2025-12-29

**Authors:** Sree Sathvik, Vijay K Silan, Srishti Singh, Ilakkiya Palaniappan

**Affiliations:** 1 Community Medicine, Bhagat Phool Singh Government Medical College for Women, Sonepat, IND

**Keywords:** condom use, content analysis, national health mission (nhm) condom use guidelines, sexual health education, youtube health information

## Abstract

Background: Correct condom use is essential for preventing sexually transmitted infections (STIs), human immunodeficiency virus (HIV), and unintended pregnancies, yet significant knowledge gaps persist, especially among young people in India. YouTube is widely used for sexual-health information, but its engagement-driven algorithm often promotes videos with high views rather than high accuracy, increasing the risk of misinformation. Limited evidence exists on the accuracy and completeness of condom-use instructions available to Indian audiences.

Objective: To analyze condom-use steps presented in popular YouTube videos in English, Hindi, and Tamil, and compare them with the National Health Mission (NHM) condom-use guidelines.

Methods: A cross-sectional content analysis was conducted on 30 YouTube videos (15 in English, eight in Hindi, seven in Tamil) identified through predefined multilingual keywords in incognito search. Videos were evaluated using a 16-point Total Score (T Score) and an 8-point Vital Score (V Score), both developed by the authors based on the NHM condom-use guidelines. Descriptive statistics were used to assess completeness, accuracy, and presentation patterns.

Results: Major instructional gaps were observed across all four steps of condom use. On average, videos mentioned 2.6 of five instructions (52%) for Step 1 (opening), 2.5 of three instructions (83.3%) for Step 2 (application), 0.5 of three instructions (16.7%) for Step 3 (during sex), and 1.7 of five instructions (34%) for Step 4 (disposal). English-language videos performed best (mean V Score: 4.7/8), followed by Hindi (4.4/8) and Tamil (3.0/8). Videos with >10 million views, those published by channels with 10,000-100,000 subscribers, and videos with >500 comments were significantly associated with higher instructional quality. No video demonstrated all eight essential steps.

Conclusion: The analyzed YouTube videos did not consistently follow the step-by-step NHM Condom Use Guidelines, with lower frequencies of instruction during the “during sex” and disposal stages and incomplete coverage even within more frequently mentioned steps. These instructional gaps mirror documented user errors, suggesting that suboptimal online content may indirectly contribute to improper condom-use practices. Improving the completeness and stage-wise consistency of videos - preferably through professional review and alignment with freely available NHM guidelines - may enhance the reliability of YouTube as a sexual-health education resource.

## Introduction

Correct and consistent condom use is essential for preventing sexually transmitted infections (STIs), including human immunodeficiency virus (HIV), as well as unplanned pregnancies. However, a substantial knowledge gap persists globally, particularly among adolescents and young adults, regarding proper condom-use steps [1]. In India, sociocultural stigma surrounding sexual health further limits open discussion, leading many young individuals to seek information from informal, often unreliable sources [2].

With the widespread availability of smartphones and the rise of digital platforms, YouTube has emerged as a major source of health-related information for young people [3,4]. Studies have shown that online content significantly influences users' health beliefs and practice [5]. However, several analyses have demonstrated that a considerable proportion of YouTube's health-related videos contain misinformation or are of poor educational quality [6,7].

YouTube’s recommendation algorithm is primarily designed to optimize user engagement, especially watch time and viewing history, rather than the factual accuracy or educational quality of the content. Thus, videos that generate higher views, clicks, and watch-time are more likely to be promoted, even if they contain misleading or incomplete information [8]. Systematic reviews have further shown that such engagement-driven systems can amplify low-quality or inaccurate content, increasing the risk of misinformation in health-related topics [9]. As a result, misleading videos on condom use may gain prominence, potentially contributing to incorrect practices, condom failure, and increased risk of STIs and unintended pregnancies.

India represents one of YouTube’s largest user bases, with over 2,580 million active internet users and more than 80% smartphone penetration [10]. Despite this, limited research exists assessing the accuracy of YouTube videos explaining condom-use steps for Indian audiences. Although YouTube has implemented measures to guide users toward authoritative sources during global health emergencies such as coronavirus disease 2019 (COVID-19), similar efforts for sexual health education, particularly for correct condom-use instructions, could be highly effective in reducing STIs, HIV, and unintended pregnancies among vulnerable populations such as adolescents.

Given these concerns, it is important to examine whether condom-use steps presented on YouTube align with evidence-based guidelines issued by national organizations such as the National Health Mission (NHM), India [11].

Therefore, this study aims to evaluate the completeness and accuracy of condom-use steps presented in popular YouTube videos in English, Hindi, and Tamil by comparing them with established national guidelines, and to examine variations and gaps in these publicly accessible videos in order to inform future improvements in digital sexual-health education.

## Materials and methods

The current study was conducted to examine disparities between evidence-based condom-use guidelines and the steps outlined in the NHM Condom Use Guidelines as presented in publicly available instructional videos on YouTube. The methodology is as follows.

Selection of keywords 

The study used specific keywords in English, Hindi, and Tamil languages to search for videos related to proper condom use on YouTube. The selected keywords were “condom use", "correct way of putting on a condom", "how to use a condom”, “how to put on a condom”, “condom ka ishtamal kaise kare”, “how to use a condom hindi”, “how to use a condom tamil”, “aanurai payanpaduthuvathu epadi”, “condom payanpaduthuvathu epadi”.

Search strategy

The search for videos was conducted on YouTube using the Google Chrome browser in incognito mode to avoid biases arising from previous search history or personalized recommendations. Searches were performed in English, Hindi, and Tamil using predefined keywords related to correct condom-use steps. Since the authors were fluent in all three languages, videos across these languages could be screened and interpreted accurately without requiring translation. Although a large number of videos appeared in the initial search results, only the top results displayed under YouTube’s default ‘relevance’ sorting were screened, as this reflects typical user behavior: viewers generally select videos from the first few pages of search results. A sample size of 30 videos was predetermined to ensure feasibility for detailed content analysis while maintaining linguistic representation. Accordingly, 15 English, eight Hindi, and seven Tamil videos that met all inclusion criteria were included in the final analysis.

Selection criteria 

The selection of videos was based on the relevance of the content to the keywords, and the length of the video was limited to less than 20 minutes. Duplicate videos, entertainment-oriented videos, and those that had fewer than 100 views were excluded to ensure a representative sample of popular videos.

Comparison with evidence-based guidelines 

The selected videos were watched and analyzed to identify the condom-use instructions presented. These instructions were compared with evidence-based condom-use guidelines, with the NHM Condom Use Guidelines adopted as the standard reference due to their relevance in the Indian context. The NHM guidelines are freely available in the public domain through the National Health Mission, Government of India website, making them an accessible and appropriate open-access standard for comparison [11].

Data extraction 

Data from each video were extracted using a structured sheet that recorded the video title, language (English, Hindi, Tamil), duration, gender of the speaker, number of views and likes, upload date, total number of videos available on the channel, and the total number of channel subscribers.

Data analysis 

Descriptive statistics (frequencies and percentages) were used to summarize the characteristics of the selected videos and the condom-use steps presented. One-way ANOVA was used to assess differences across videos for variables, including language, video duration, time since upload, number of views, number of likes, number of comments, total number of videos on the channel, and total number of channel subscribers. An unpaired Student's t-test was conducted to evaluate differences in instruction scores based on the gender of the speaker. A p-value of <0.05 was considered statistically significant.

Scoring for analysis 

According to the NHM condom-use guidelines, a total of 16 recommended instructions were identified across four steps. Each instruction was assigned one point, resulting in a maximum achievable score of 16, termed the T Score (Total Score) for each video.

From these 16 instructions, the authors identified eight instructions as critically important, representing the minimum essential steps required to prevent condom failure and ensure effective use. These eight items were selected through a consensus method by two public health experts who independently evaluated instructions for their direct impact on preventing condom breakage, slippage, or incorrect application, with inter-reviewer agreement achieved through consensus discussion to resolve discrepancies. The maximum score attainable for these essential items was 8, termed the V Score (Vital Score).

The V Score includes important instructions as follows. Under Step 1, "Use a new condom each time you have sex," "Remove condom carefully," and "DO NOT use fingernails, teeth, or sharp objects to open the package or remove condom." In Step 2, "Squeeze closed the top end of the condom to make sure no air is inside (which can make it break)." In Step 3, "Once sperm has been released into the condom (ejaculation), withdraw the erect penis and HOLD the condom in place on the penis." Finally, in Step 4, "Knot the end of the used condom," "Place in tissue or a bag before throwing it in the dustbin," and "DO NOT flush condoms down the toilet. It will block the system."

Ethical issues

Since this study involved the analysis of publicly available materials on YouTube, no direct human subjects were involved, and no identifiable information was collected. Additionally, no biological samples were collected from any participants. Therefore, this study did not require ethical approval from an institutional review board. However, it was essential to ensure that the search and analysis of videos were conducted responsibly and respectfully. The researchers ensured that they did not engage in any activity that could be deemed inappropriate or violate any community guidelines or laws. Moreover, the videos were analyzed objectively, without any personal biases or prejudices. The researchers also ensured that any data collected during the study were stored securely and anonymously. The findings of this study will be disseminated in a way that protects the privacy and confidentiality of the individuals featured in the videos. Hence, we did not seek ethical approval for this study and request a waiver of ethics.

## Results

From our current study we found that, in Step 1 (Opening the package), only 12 videos (40%) advised using a new condom each time, while checking expiry and package integrity was the most frequently mentioned instruction (23 videos, 76.7%). Only five videos (16.7%) demonstrated pushing the condom to one side before tearing the packet, and instructions on careful removal and avoiding sharp objects were each shown in 19 videos (63.3%). Step 2 (Putting it on) had comparatively better coverage, with 25 videos (83.3%) demonstrating squeezing the tip to expel air, 21 videos (70%) showing correct placement on the erect penis, and 28 videos (93.3%) illustrating unrolling the condom down the shaft. Step 3 (During sex) was the least adequately presented: ensuring the condom stays in place appeared in only two videos (6.7%), advice to replace the condom if it slips off was mentioned in three videos (10%), and guidance to hold the condom at the base during withdrawal was shown in 11 videos (36.7%). Step 4 (Disposal) was also incompletely covered, with only eight videos (26.7%) advising removal after complete withdrawal and just one video (3.3%) cautioning against contact with the partner’s body during removal. Knotting the condom was mentioned in 16 videos (53.3%) and wrapping it before discarding in 14 videos (46.7%) (Table 1).

**Table 1:** Distribution of Data Across Instructions Under Each Step of the National Health Mission (NHM) Condom Use Guidelines (N = 30)

Steps	No. of videos that mentioned the step N (%)
Step 1 – Open package	
Use a new condom each time you have sex	12 (40)
Check that it has not expired and that the packaging has no holes by pressing the pack between your fingers	23 (76.7)
Push condom to one side of package to allow room to tear open other side	5 (16.7)
Remove condom carefully	19 (63.3)
DO NOT use fingernails, teeth, or sharp objects to open package or remove condom	19 (63.3)
Use a new condom each time you have sex	12 (40)
Check that it has not expired and that the packaging has no holes by pressing the pack between your fingers	23 (76.7)
Step 2 – Put it on	
Squeeze closed top end of condom to make sure no air is inside (can make it break)	25 (83.3)
Place condom over top of erect penis	21 (70)
With other hand, unroll condom gently down the full length of your penis (one hand still squeezing top end)	28 (93.3)
Step 3 – During sex	
Make sure condom stays in place	2 (6.7)
If it comes off, withdraw your penis and put on a new condom before intercourse continues	3 (10)
Once sperm has been released into condom (ejaculation), withdraw the erect penis and HOLD the condom in place on penis	11 (36.7)
Step 4 – Dispose of condom	
Remove condom ONLY when penis is fully withdrawn	8 (26.7)
Keep both penis and condom clear from contact with your partner's body	1 (3.3)
Knot the end of the used condom	16 (53.3)
Place in tissue or bag before throwing it in dustbin	14 (46.7)

Our analysis showed that, on average, only 2.6 of the five recommended instructions for Step 1 (52%) and 2.5 of the three instructions for Step 2 (83.3%) were presented in the videos. Step 3 was the most poorly represented, with only 0.5 of the three recommended instructions mentioned. For Step 4, an average of 1.7 of the five instructions (34%) was covered. Overall, the analysed YouTube videos included only 7.3 of the 16 essential instructions (45.6%) from the NHM Condom Use Guidelines (Table 2).

**Table 2:** Distribution of Data Across Steps of the National Health Mission (NHM) Condom-Use Guidelines (N = 30)

Steps	Maximum score	Average score obtained (%)
Step 1 – Open package	5	2.6 (52)
Step 2 – Put it on	3	2.5 (83.3)
Step 3 – During sex	3	0.5 (16.7)
Step 4 – Dispose of condom	5	1.7 (34)
Overall	16	7.3 (45.6)

English-language videos had the highest mean V Score (4.7), followed by Hindi (4.4) and Tamil (3.0) (p = 0.118), and videos with male and female speakers showed similar scores (4.2 vs. 4.3; p = 0.851). Video duration (less than two minutes: 4.4; two to seven minutes: 4.2; more than seven minutes: 4.1; p = 0.973) and time since uploading (more than five years: 4.6; one to five years: 4.4; less than one year: 3.9; p = 0.709) showed no significant differences. High-view videos (>10 million) had the highest V Score (6.3) compared to moderate (3.6) and low-view videos (4.6) with a significant association (p = 0.033). Videos with more likes showed higher V Scores (5.3 for >10,000 likes), though not statistically significant (p = 0.081), and comment categories also showed no significant difference (p = 0.277). Channel size by number of uploaded videos did not influence V Score (p = 0.899), while subscriber count showed a significant association, with moderate-subscriber channels (10,000-100,000) scoring highest (5.7) compared to big (4.5) and small channels (3.3) (p = 0.022) (Table 3).

**Table 3:** Distribution of Data Across Determinants and Corresponding V Scores (N = 30) All comparisons across groups with more than two categories (e.g., language, video duration, time since uploading, views, likes, comments, number of videos in the channel, and number of channel subscribers) were analyzed using one-way Analysis of Variance (ANOVA). For variables with only two categories, marked with an asterisk (*)—i.e., gender of the speaker—an unpaired Student's t-test was applied. V Score = Vital Score

Variables	No. of Videos N (%)	Average V Score (out of 8)	Test Statistic	p value
3.1 Speaker Details				
Language				
English	15 (50)	4.7	F = 2.317	0.118
Hindi	8 (27)	4.4		
Tamil	7 (23)	3		
Gender of the speaker*				
Male	17 (56.7)	4.2	t = 0.189	0.851
Female	13 (43.3)	4.3		
3.2 Video-Related Details				
Duration of the video				
Less than 2 minutes	5 (17)	4.4	F = 0.027	0.973
2 to 7 minutes	18 (60)	4.2		
More than 7 minutes	7 (23)	4.1		
Time since uploading				
> 5 years	7 (23)	4.6	F = 0.349	0.709
1 to 5 years	9 (30)	4.4		
<1year	14 (47)	3.9		
Views				
High (>10 million)	3 (10)	6.3	F = 3.888	0.033
Moderate (100,000 -10 million)	16 (53)	3.6		
Low (< 100,000)	11 (37)	4.6		
Likes				
High (>10,000)	10 (33.3)	5.3	F = 2.760	0.081
Moderate (1,000 to 10,000)	11 (36.7)	4		
Low (<1,000)	9 (30)	3.4		
Comments				
High (>500)	5 (17)	5.6	F = 1.358	0.277
Moderate (50 - 500)	8 (27)	4.1		
Low (<50)	10 (33)	3.6		
Turned off	7 (23)	4.3		
3.3 Channel-Related Details				
No. of videos on the channel				
Big Channels (>500)	8 (27)	4.1	F = 0.106	0.899
Moderate (50 - 500)	14 (47)	4.1		
Small Channels (<50)	8 (27)	4.5		
No. of subscribers				
Big Channels (>100,000)	11 (37)	4.5	F = 4.427	0.022
Moderate Channels (10,000 – 100,000)	6 (20)	5.7		
Small Channels (<10,000)	13 (43)	3.3		

English-language videos had the highest mean T Score (8.5), followed by Hindi (7.0) and Tamil (4.9), with a significant difference (p = 0.027). Male and female speakers showed similar T Scores (7.1 vs. 7.5; p = 0.682). Video duration showed no significant variation, with mean T Scores of 7.4 (less than two minutes), 6.9 (two to seven minutes), and 5.8 (more than seven minutes) (p = 0.804). Time since upload also showed no significant difference (p = 0.478), with T Scores of 8.3 (more than five years), 7.6 (one to five years), and 6.6 (less than one year). High-view videos (>10 million) had the highest T Score (10.3) compared to moderate (6.4) and low-view videos (7.6), though nonsignificant (p = 0.118). Videos with high likes (>10,000) had significantly higher T Scores (9.2) than moderately liked (7.0) and low-liked (5.6) videos (p = 0.046). Comment categories showed no significant differences (p = 0.137). Channel size by number of uploaded videos showed no association with T Scores (p = 0.687), whereas subscriber count showed a significant difference (p = 0.046), with moderate-subscriber channels (10,000-100,000) scoring highest (9.2), followed by big channels (8.0), and small channels (<10,000) showing the lowest scores (5.8) (Table 4).

**Table 4:** Distribution of Data Across Determinants and Corresponding T Scores (N = 30) All comparisons across groups with more than two categories (e.g., language, video duration, time since uploading, views, likes, comments, number of videos in the channel, and number of channel subscribers) were analyzed using one-way Analysis of Variance (ANOVA). For variables with only two categories, marked with an asterisk (*)—i.e., gender of the speaker—an unpaired Student's t-test was applied. T Score = Total Score

Variables	No. of Videos N (%)	Average T Score (out of 16)	Test Statistic	p value
4.1 Speaker Details				
Language				
English	15 (50)	8.5	F = 4.145	0.027
Hindi	8 (27)	7		
Tamil	7 (23)	4.9		
Gender of the speaker*				
Male	17 (56.7)	7.1	t = 0.415	0.682
Female	13 (43.3)	7.5		
4.2 Video-Related Details				
Duration of the video				
Less than 2 minutes	5 (17)	7.4	F = 0.220	0.804
2 to 7 minutes	18 (60)	6.9		
More than 7 minutes	7 (23)	5.8		
Time since uploading				
> 5 years	7 (23)	8.3	F = 0.478	0.478
1 to 5 years	9 (30)	7.6		
<1year	14 (47)	6.6		
Views				
High (>10 million)	3 (10)	10.3	F = 2.318	0.118
Moderate (100,000 -10 million)	16 (53)	6.4		
Low (< 100,000)	11 (37)	7.6		
Likes				
High (>10,000)	10 (33.3)	9.2	F = 3.462	0.046
Moderate (1,000 to 10,000)	11 (36.7)	7		
Low (<1,000)	9 (30)	5.6		
Comments				
High (>500)	5 (17)	10	F = 2.013	0.137
Moderate (50 - 500)	8 (27)	6.9		
Low (<50)	10 (33)	6.1		
Turned off	7 (23)	7.4		
3.3 Channel-Related Details				
No. of Videos in the Channel				
Big Channels (>500)	8 (27)	7.4	F = 0.381	0.687
Moderate (50 - 500)	14 (47)	6.8		
Small Channels (<50)	8 (27)	8		
No. of subscribers				
Big Channels (>100,000)	11 (37)	8	F = 3.463	0.046
Moderate Channels (10,000 – 100,000)	6 (20)	9.2		
Small Channels (<10,000)	13 (43)	5.8		

Importantly, none of the videos demonstrated all the recommended steps as outlined in the NHM Condom Use Guidelines, indicating that even the most fundamental instructions were incompletely presented across videos.

## Discussion

Step 1

In our study, only 2.6 out of five instructions (52%) related to Step 1 - Opening the package were demonstrated in the analysed videos. Key instructions such as using a new condom each time were shown in only 12 videos (40%), while checking the expiry date and package integrity appeared in 23 videos (76.7%). Guidance on removing the condom carefully to avoid damage was demonstrated in 19 videos (63%), and pushing the condom to one side before tearing the package - an important step to prevent accidental tearing - was shown in only five videos (16.7%). These gaps could contribute to common condom-use errors reported in the literature. For example, a previous study found that 83% of users did not use a new condom when switching between different sexual acts [12]. Another study reported that 37% of participants knowingly used expired condoms and 79% failed to check for possible damage [13]. Although videos in our study frequently emphasized avoiding sharp objects and removing the condom carefully, evidence shows that 74-82.7% of users have still reported condom breakage or damage, and about 11% admitted using sharp objects to open the pack [12,14]. These findings highlight that incomplete or inconsistent instructional content in publicly available videos may contribute to persistent user-level errors observed in empirical studies.

Step 2

In our study, 2.5 out of three instructions (83.3%) related to Step 2 - Put it on were included in the analyzed videos, making it the most consistently conveyed step across all content. Despite this comparatively high level of accurate instruction, user-level errors during condom application remain widely reported in the literature, indicating that the presence of correct information alone may not translate into correct practice. One study reported that 70.6% of participants did not squeeze the tip of the condom before application, and 25% did not roll the condom down to the base of the penis [15]. Another study also documented frequent errors such as the condom being removed during intercourse, placing the condom inside out, improper withdrawal, failure to squeeze out air from the tip, and not leaving adequate space at the reservoir tip [14].

Step 3

In our study, only 0.5 out of three instructions (16.7%) related to Step 3 - During sex were conveyed in the analyzed videos, making this the least addressed and most neglected step of condom use. Critical instructions such as ensuring that the condom stays in place were mentioned in only two videos (6.7%), and advising users to put on a new condom if it comes off was included in just three videos (10%), despite their importance for effective and safe condom use. Although YouTube is not the sole source of sexual health information, the incomplete communication of these instructions highlights gaps in online educational content and, while this study does not assess user behavior or outcomes, may indirectly contribute to persistent user-level errors reported in the population. Studies have reported that 36.9% of users experienced condom breakage or slippage, and 19.7% reported reusing condoms [13,15]. Furthermore, multivariate logistic regression findings from a study indicate that factors such as applying the condom on the wrong side, putting it on after intercourse has begun, removing it during sex, and condom slippage significantly predict condom breakage [16]. These patterns highlight the potential consequences of inadequate instructional content during the ‘during sex’ phase.

Step 4

In our study, only 1.7 out of five instructions (34%) related to Step 4 - Dispose of the condom were conveyed in the analyzed videos. Essential steps such as removing the condom only after fully withdrawing the penis were mentioned in just eight videos (26.7%), and ensuring that the condom and penis do not come into contact with the partner’s body was included in only one video (3.3%). Instructions such as tying a knot at the end of the condom were shown in 16 videos (53.3%), wrapping it in tissue or placing it in a bag before disposal appeared in 14 videos (46.7%), and guidance on avoiding flushing it down the toilet was rarely emphasized. Inadequate or inconsistent communication of these disposal-related steps may undermine some of the public health benefits associated with correct condom use, particularly in terms of hygiene, environmental safety, and reducing accidental exposure risks. For instance, one study reported that 49.4% of users did not hold the base of the penis during withdrawal, and 36.9% experienced condom breakage or slippage [15]. Additionally, evidence from Ethiopia shows a more than twofold increase in STI (hepatitis B) risk among individuals exposed to medical waste, and this risk is further exacerbated by socio-economic vulnerability [17,18]. These findings are particularly relevant in countries like India, where large segments of the population reside in rural and low-income communities, and where improper condom disposal practices may amplify existing health risks.

These findings collectively highlight that incomplete or inconsistent instructional content in publicly available videos may contribute to persistent user-level errors observed in empirical studies. Even when certain steps are more frequently demonstrated, correct technique does not always translate into practice, suggesting that gaps in understanding and application remain. Inadequate or poorly communicated instructions, particularly during critical phases such as the ‘during sex’ step, may therefore have significant implications for condom effectiveness and overall sexual health outcomes.

YouTube as a source of health-related information

Our study assessing YouTube videos on condom use demonstrates that while the platform has strong potential as a health-education tool, critical gaps exist in the quality and completeness of instructional content. Consistent with a study done by Prybutok G (2013), YouTube can effectively improve knowledge and influence healthy behaviors; however, more recent evaluations of technical health skills highlight limitations [19]. Pamukcu M and Izci Duran T (2021) found that self-injection videos, though often informative, varied in quality, and viewers could not reliably distinguish high-quality content [20]. Similarly, Shukla A (2021) reported that only 44.1% of popular COVID‑19 videos were educational and that viewer engagement did not necessarily reflect instructional completeness, while Ramadhani et al. (2021) observed that halitosis videos uploaded by non-professionals were prevalent and frequently of low reliability [21,22]. In line with these findings, our analysis revealed that YouTube condom-use videos frequently omit critical step-by-step instructions, particularly during intercourse and disposal, which are essential for correct technique. These studies collectively indicate that YouTube’s effectiveness as a health-education platform is limited when content is incomplete, technically inaccurate, or presented by non-professionals.

Our study shows that YouTube videos on condom use frequently omit critical step-by-step instructions, particularly during intercourse and disposal, despite the platform’s strong reach and educational potential. Stepwise analysis revealed that only 2.6 out of five instructions (52%) for opening, 2.5 out of three instructions (83.3%) for application, 0.5 out of three instructions (16.7%) for the “during sex” phase, and 1.7 out of five instructions (34%) for disposal were demonstrated in the videos, highlighting substantial gaps in essential condom-use guidance. These gaps align with documented user errors in empirical studies [12-14,16]. Comparisons with other health-related YouTube studies indicate that while the platform can improve knowledge, technical completeness and reliability vary widely [19-22]. To maximize its effectiveness for sexual health education, future videos should be professionally produced, comprehensive, stepwise, and visually demonstrative, ensuring reliability and accessibility. Such improvements could enhance proper condom-use practice and reduce persistent user errors, fulfilling YouTube’s potential as a health-education platform.

The study’s strengths include a systematic, multilingual search strategy (English, Hindi, Tamil) using specific keywords to capture a representative sample of YouTube videos on condom use. Videos were analysed stepwise, covering all four critical stages of condom use - opening, application, during sex, and disposal - and were compared against evidence-based guidelines from the National Health Mission, India. The study introduced quantitative scoring metrics (T Score) to objectively assess the completeness and quality of instructional content, and additionally used V Scores based on the most vital instructions from the T Score. The use of descriptive statistics and structured data extraction ensured clarity and reproducibility in identifying instructional gaps. The study also considered accessibility and usability by including videos across different platforms (desktop and mobile) and accounting for age-restricted content.

This study has several limitations. First, only YouTube videos were assessed; instructional content available on other social media platforms or offline resources was not included. Second, the analysis was restricted to popular videos (duration <20 minutes and >100 views), and only those ranked highest by YouTube’s relevance algorithm, which may have excluded niche, high-quality, or recently uploaded content that did not meet these criteria. Third, only 30 videos were included in the final analysis, which may limit the generalizability of the findings. Fourth, viewer comprehension and actual behaviour change after watching these videos were not evaluated, preventing conclusions about the real-world impact of the instructional content. Finally, language coverage was restricted to English, Hindi, and Tamil, potentially omitting relevant instructional videos in other regional languages.

## Conclusions

The videos analysed in our study did not consistently or comprehensively follow the step-by-step instructions outlined in the NHM Condom Use Guidelines. Key stages, particularly the “during sex” and disposal steps, were frequently omitted, and even the more commonly demonstrated steps lacked complete coverage of essential sub-instructions. These findings indicate substantial gaps in the instructional completeness of publicly available condom-use videos on YouTube. To improve the educational value of such content, future videos should provide complete, guideline-based instructions, ideally produced or reviewed by health professionals, and include links to the freely available NHM Condom Use Guidelines on the Government of India’s National Health Mission website. Platform-level quality checks for health-related instructional videos may further ensure accuracy. Enhancing completeness and consistency will help viewers receive correct and reliable information, supporting safer sexual-health behaviours.
